# Giant palpebral hidrocystoma

**DOI:** 10.11604/pamj.2025.52.115.49927

**Published:** 2025-11-17

**Authors:** Youssef Zemmez, Naoufal Hjira

**Affiliations:** 1 Department of Dermatology-Venereology, Mohammed V Military Training Hospital, Rabat, Morocco

**Keywords:** Hidrocystoma, giant, surgery

## Image in medicine

Hidrocystoma belongs to benign tumors of sweat glands. It varies in size from a few millimeters to 1,5 cm in rare cases. We report an atypical case of giant hidrocystoma of the eyelid. This is a 52-year-old female patient with no particular medical history who presented at a dermatology clinic with skin lesions on both eyelids that had been developing for twenty years. Dermatological examination revealed multiple budding tumor lesions that were soft and painless on palpation, brownish-yellow in color, affecting the upper left and right eyelids as well as the inner canthi on both sides. The patient was referred to plastic surgery for an excisional biopsy, and histology confirmed the diagnosis of multiple hydrocystomas. Palpebral hidrocystomas are benign tumors also known as cystic apocrine adenoma, cyst of sweat gland, apocrine retention cyst or cyst of Moll. They originate from eccrine or apocrine sweat glands and often occur on the face and the eyelids. Other atypical locations such as the chest, the shoulders and the foreskin have been reported. Hidrocystoma is a small translucent, shiny cyst. It appears as single or multiple cystic lesions.

**Figure 1:**
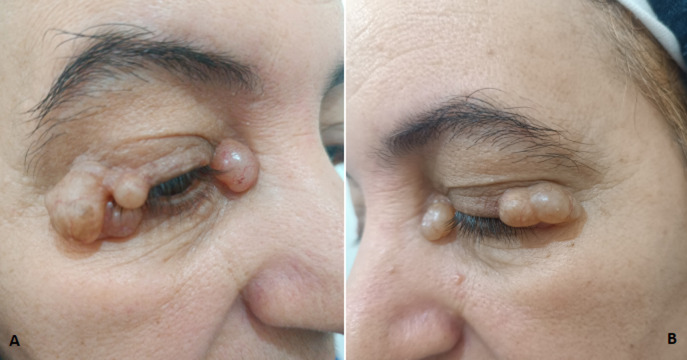
A) right eyelid hidrocystoma; B) left eyelid hidrocystoma

